# Supplementary material for: A release of local subunit conformational heterogeneity underlies gating in a muscle nicotinic acetylcholine receptor
Source: Nat Commun. 2024 Feb 27;15:1803. doi: 10.1038/s41467-024-46028-x (PMC10899235; doi:10.1038/s41467-024-46028-x)
Supplement: Supplementary file 1 — Supplementary Information [file 41467_2024_46028_MOESM1_ESM.pdf]

Supplemental information

A release of local subunit conformational heterogeneity underlies gating in a muscle nicotinic acetylcholine receptor.

Mackenzie J Thompson, Farid Mansoub Bekarkhanechi, Anna Ananchenko, Hugues Nury, and John E Baenziger[1]

[1]Department of Biochemistry, Microbiology, and Immunology, University of Ottawa, Ottawa ON, K1H 8M5, Canada
[2]Université Grenoble Alpes, CNRS, CEA, IBS, F-38000 Grenoble, France.

To whom correspondence should be addressed: John E. Baenziger, Department of Biochemistry, Microbiology, and Immunology, University of Ottawa, 451 Smyth Rd. Ottawa, ON, K1H 8M5, Canada, Tel.: (613) 562-5800 x8222; Fax.: (613) 562-5440; E-mail: John.Baenziger@uottawa.ca.

Tables of contents

Table S1. Functional effects of mutations to the ECD triad and residues in the M2-M3 loop of the Torpedo versus human adult muscle nAChR. 2
Table S2. Functional effects of mutations to β8-β9/β10-M1 and M2-M3 of the Torpedo nAChR. 3
Table S3. Energetic couplings between individual residues at the β8-β9/β10-M1 - M2-M3 interfaces. 4
Table S4. Non-specific bumping of residues at the $\alpha_\gamma/\alpha_\delta$ M2-αM3 - γ/δ β8-β9 loops on nAChR. 5
Table S5. Subunit specific effects of mutating residues contributing to conserved tripartite salt bridges at the ECD – TMD interfaces. 6
Figure S1. Sensitivities of EC_{50} versus θ to changes in channel function. 7
Figure S2. The quadruple Asn αM2-M3 mutant stabilizes the resting state. 18
Figure S3. The interdependence between γGly182/δGly188 and the αγ/αδ M2-M3 loops is mediated by a backbone hydrogen bond with αSer268. 17
Figure S4. Raw TEVC traces used to plot dose-response curves. 9
Supplemental Discussion of the conformation adopted by the ECD of each subunit in apo and agonist bound forms of the Torpedo nAChR 19
References 24

Table S1. Functional effects of mutations to the ECD triad and residues in the M2-M3 loop of the *Torpedo* versus human adult muscle nAChR.

	Torpedo nAChR[a]				Human adult nAChR[a]			
Mutant	EC_{50} (μM)	Hill slope	n	Fold change	EC_{50} (μM)	Hill slope	N	Fold change
WT	9.40 ± 1.82	1.78 ± 0.35	87	-	7.98 ± 1.75	1.87 ± 0.32	53	-
αE45R	0.600 ± 0.316	1.39 ± 0.21	8	15.7 gain	1.40 ± 0.39	1.43 ± 0.31	8	5.71 gain
αE45A	25.1 ± 5.5[b]	1.83 ± 0.31	8	2.67 loss	22.3 ± 4.0[b]	1.85 ± 0.31	8	2.79 loss
αE45K	NR[c]				3.63 ± 0.61	1.77 ± 0.45	8	2.20 gain
αV46A	108 ± 21[b]	2.00 ± 0.41	8	11.5 loss	69.4 ± 13.5[b]	1.38 ± 0.35	8	8.70 loss
αR209Q	NE[d]				NR[c]			
αR209E	NE[d]				NR[c]			
αP272G	27.1 ± 4.9[b]	1.63 ± 0.15	10	2.88 loss	37.9 ± 9.4[b]	1.82 ± 0.37	8	4.75 loss
αE45R+αR209Q	9.16 ± 2.47	1.92 ± 0.40	8	1.03 gain	2.50 ± 0.35	2.18 ± 0.22	6	3.19 gain
αE45R+αR209E	9.80 ± 2.23	1.72 ± 0.09	8	1.04 loss	NR[c]			
αE45A+αV46A	NR[c]				7.32 ± 0.73	2.26 ± 0.20	9	1.10 gain
αE45A+αP272G	55.8 ± 8.2[b]	1.49 ± 0.18	8	5.93 loss	NR[c]			
αV46A+αP272G	142 ± 33[b]	2.29 ± 0.50	8	15.1 loss	38.0 ± 9.4[b]	1.56 ± 0.50	8	4.76 loss
αE45A+αV46A+ αP272G	NR[c]				NR[c]			

[a]Measurements performed 2-4 days after cRNA injection (V_{hold} = -60 mV). Error values represented as standard deviation
[b]$p < 0.001$ relative to WT via one-way ANOVA followed by Dunnet's post hoc test. *Torpedo* DF=144. *Torpedo* F_{EC50}=294.9. *Torpedo* F_{Hill}=5.657. Human adult DF=107. Human adult F_{EC50}=185.8. Human adult F_{Hill}=6.736. Exact p values can be found in a supplemental file.
[c]No response (NR). No significant agonist induced current observed up to 4 day after cRNA injection
[d]No expression (NE). No significant expression or agonist induced current observed up to 4 day after cRNA injection

Table S2. Functional effects of mutations to β8-β9/β10-M1 and M2-M3 of the *Torpedo* nAChR.

Mutant[a]	EC_{50} (μM)	Hill slope	n	Fold change
WT	9.40 ± 1.8	1.78 ± 0.35	87	
α7 Loop F transplants				
δ(^{178}IIDPEA^{183} → ^{178}DISG--^{181})	16.7 ± 9.8[b]	0.72 ± 0.19[b]	8	1.78 loss
γ(^{173}HIDPED^{178} → ^{173}DISG--^{176})	54.4 ± 3.7[b]	1.41 ± 0.13	8	5.78 loss
δ+γ(loop F → ^{173}DISG--^{176})	98.3 ± 22.7[b]	1.71 ± 0.40	5	10.5 loss
M2-M3 of α subunit				
αS268P	9.67 ± 1.69	1.81 ± 0.41	8	1.03 loss
αS266A+αT267A	31.4 ± 3.3[b]	1.99 ± 0.12	8	3.34 loss
αS268A+αS269A	0.857 ± 0.342[b]	1.55 ± 0.32	8	11.0 gain
β8-β9 and β10-M1 of γ and δ subunit				
γN181A	7.49 ± 1.23	1.95 ± 0.16	8	1.26 gain
γN181D	1.65 ± 0.56[b]	1.91 ± 0.50	8	5.69 gain
γN181Y	9.29 ± 2.08	2.00 ± 0.31	10	1.01 gain
δN187D	5.82 ± 0.60	1.84 ± 0.27	9	1.62 gain
γW184A	10.4 ± 1.6	2.07 ± 0.34	8	1.10 loss
γT185A	7.64 ± 2.06	1.92 ± 0.35	8	1.23 gain
γT185D	4.60 ± 1.16	1.64 ± 0.24	8	2.04 gain
δE191A	11.6 ± 2.1	1.83 ± 0.22	9	1.23 loss
δE191K	17.6 ± 4.8[b]	2.09 ± 0.27	9	1.87 loss
γI215A	22.6 ± 4.5[b]	1.58 ± 0.19	8	2.41 loss
γR217A	10.5 ± 1.5	2.05 ± 0.34	8	1.11 loss
γR217W	NE[c]			
γR217E	NE[c]			
γR217K	NE[c]			
δR223A	7.02 ± 1.37	1.60 ± 0.26	8	1.33 gain
δR223W	5.73 ± 1.06	1.82 ± 0.59	8	1.63 gain
δR223E	5.95 ± 2.09	1.99 ± 0.42	9	1.57 gain
δR223K	16.8 ± 13.3[b]	1.41 ± 0.57	7	1.78 loss
δR223Q	7.36 ± 2.50	1.32 ± 0.48	8	1.28 gain
γK218R	5.14 ± 1.36	1.88 ± 0.24	8	1.83 gain
γK218E	2.34 ± 0.49[b]	1.95 ± 0.21	8	4.03 gain
γK218M	2.28 ± 0.33[b]	2.11 ± 0.51[b]	8	4.13 gain
δK224E	4.84 ± 0.65	1.84 ± 0.30	8	1.94 gain
δK224M	3.91 ± 0.61	2.61 ± 0.65	8	2.40 gain
γL220F	2.87 ± 0.57[b]	1.94 ± 0.42	8	3.27 gain
γY222A	13.9 ± 1.3	1.83 ± 0.32	8	1.48 loss
δY228A	18.6 ± 3.3[b]	1.64 ± 0.22	8	2.00 loss
γK272A	12.4 ± 2.4	1.80 ± 0.32	8	1.32 loss
δR277A	10.8 ± 1.5	1.67 ± 0.23	8	1.15 loss

[a]Measurements performed 2-4 days after cRNA injection (V_{hold} = -60 mV). Error values represented as standard deviation
[b]$p < 0.001$ relative to WT via one-way ANOVA followed by Dunnet's post hoc test. DF=312. F_{EC50}=127.9. F_{Hill}=5.405. Exact p values can be found in a supplemental file.

Table S3. Energetic couplings between individual residues at the β8-β9/β10-M1 - M2-M3 interfaces.

Mutant	EC_{50} (μM)	Hill slope	n	Fold change	Predicted[b]	Ω	ΔΔG (kJ/mol)[d]
αS266A+γG182A	44.5 ± 4.9[b]	2.47 ± 0.56[b]	8	4.74 loss	11.0 loss	0.43	-2.09 ± 0.64
αS266A+γG182I	7.91 ± 3.21	1.98 ± 0.29	9	1.19 gain	1.34 loss	0.63	-1.15 ± 0.58
αS266A+γG182S	32.0 ± 2.9[b]	1.85 ± 0.16	9	3.41 loss	6.87 loss	0.50	-1.74 ± 0.51
αS266A+δG188A	46.2 ± 6.2[b]	2.18 ± 0.32	9	4.91 loss	9.06 loss	0.54	-1.52 ± 0.52
αS266A+γE183A	36.7 ± 5.84[b]	1.87 ± 0.27	10	3.90 loss	7.47 loss	0.52	-1.61 ± 0.59
αS266A+γK218A	15.7 ± 4.5[b]	1.76 ± 0.42	8	1.67 loss	1.12 loss	1.48	+0.98 ± 0.47
αS266A+γK272A	37.4 ± 14.4[b]	1.81 ± 0.42	9	3.98 loss	5.83 loss	0.68	-0.95 ± 0.48
αT267A+γG182A	32.5 ± 3.4[b]	2.16 ± 0.46	8	3.45 loss	7.25 loss	0.48	-1.83 ± 0.50
αT267A+γG182I	16.2 ± 2.2[b]	1.69 ± 0.19	8	1.73 loss	1.13 gain	1.96	+1.67 ± 0.49
αT267A+γG182S	28.1 ± 4.7[b]	1.85 ± 0.15	8	2.99 loss	4.52 loss	0.66	-1.03 ± 0.30
αT267A+δG188A	33.2 ± 2.8[b]	1.93 ± 0.23	9	3.53 loss	5.96 loss	0.59	-1.30 ± 0.39
αT267A+γK218A	10.1 ± 4.4	2.07 ± 0.46	8	1.07 loss	1.35 gain	1.45	+0.92 ± 0.52
αS268A+γE183A	4.54 ± 1.12	1.60 ± 0.32	8	2.07 gain	2.52 gain	1.22	+0.49 ± 0.20
αS268A+γK218A	0.911 ± 0.134[b]	1.89 ± 0.53	8	10.3 gain	16.8 gain	1.63	+1.21 ± 0.48
αS268A+γK218E	0.524 ± 0.079 [b]	2.24 ± 0.38	8	17.9 gain	17.2 gain	0.96	-0.11 ± 0.04
αS268A+δK224A	0.864 ± 0.168[b]	1.79 ± 0.40	12	10.9 gain	9.46 gain	0.87	-0.35 ± 0.13
αS268A+γL220A	1.57 ± 0.33[b]	1.80 ± 0.19	9	5.98 gain	4.54 gain	0.76	-0.69 ± 0.28
αS269A+γK218A	1.02 ± 0.12[b]	1.99 ± 0.33	8	9.19 gain	9.67 gain	1.05	+0.13 ± 0.05
αS269A+γK218E	0.652 ± 0.168[b]	2.51 ± 0.93[b]	8	14.4 gain	9.88 gain	0.68	-0.94 ± 0.37
αS269A+δK224A	1.49 ± 0.44[b]	1.59 ± 0.33	8	6.29 gain	5.44 gain	0.87	-0.36 ± 0.15

[a]Measurements performed 2-4 days after cRNA injection (V_{hold} = -60 mV). Error values represented as standard deviation

[b]Predicted fold change if both individual mutants influence function independently

[c]$p < 0.001$ relative to WT via one-way ANOVA followed by Dunnet's post hoc test. DF=237. F_{EC50}=180.6. F_{Hill}=3.562. Exact p values can be found in a supplemental file.

[d]An energetic coupling is calculated from EC_{50} values to facilitate comparisons. Energy values provided are not quantitative (see methods and Figure S1).

Table S4. Non-specific bumping of residues at the $\alpha_\gamma/\alpha_\delta$ M2-αM3 - γ/δ β8-β9 loops on nAChR.

	Dose-response[a]				[^{125}I]-α-BTX[b]
Mutant	EC_{50} (μM)	Hill slope	n	Fold change	Normalized CPM
WT	9.40 ± 1.82	1.78 ± 0.35	87	-	1.00 ± 0.11
	αM2-M3 bulky substitutions				
αS266N+T267N+S268N+S269N	NR[c]				1.38 ± 0.50[e]
αS266D+T267D+S268D+S269D	NE[d]				0.06 ± 0.03[f]
αS266W+T267W+S268W+S269W	NE[d]				0.05 ± 0.02[f]
Mock	-				0.07 ± 0.03
	γG182/δG188 substitutions				
Mutant	EC_{50} (μM)	Hill slope	n	Fold change	Residue Volume (Å^3)
γG182A	23.4 ± 2.8[f]	1.53 ± 0.26	8	2.49 loss	90.1
γG182S	14.6 ± 1.4[f]	1.81 ± 0.33	8	1.56 loss	94.2
γG182V	3.70 ± 0.52[f]	1.93 ± 0.36	8	2.54 gain	139.1
γG182E	4.99 ± 0.94[f]	1.81 ± 0.23	9	1.89 gain	140.8
γG182Q	5.71 ± 1.4[f]	2.01 ± 0.37	8	1.65 gain	149.4
γG182I	2.84 ± 0.39[f]	1.70 ± 0.03	8	3.31 gain	164.9
γG182M	13.3 ± 1.7[f]	1.70 ± 0.22	10	1.42 loss	167.7
γG182R	2.82 ± 0.52[f]	1.90 ± 0.31	9	3.33 gain	192.8
γG182W	3.47 ± 0.47[f]	1.76 ± 0.17	9	2.71 gain	231.7
δG188A	19.3 ± 3.5[f]	1.59 ± 0.20	8	2.05 loss	90.1
δG188S	16.3 ± 3.0[f]	1.60 ± 0.11	8	1.73 loss	94.2
δG188I	3.46 ± 1.53[f]	1.43 ± 0.28	8	2.72 gain	164.9
γG182A+δG188A	31.0 ± 2.1[f]	1.68 ± 0.30	8	3.30 loss	
γG182I+δG188I	NE[c]				

[a]Measurements performed 2-4 days after cRNA injection (V_{hold} = -60 mV). Error values represented as standard deviation
[b]Measurements performed 2 days after cRNA injection on a minimum of 10 oocyes. Error values represented as standard deviation
[c]No response (NR). No significant agonist induced current observed up to 4 day after cRNA injection
[d]No response (NE). No significant agonist induced current observed up to 4 day after cRNA injection
[e]$p < 0.001$ relative to mock injected oocytes via one-way ANOVA followed by Dunnet's post hoc test
[f]$p < 0.001$ relative to WT via one-way ANOVA followed by Dunnet's post hoc test. DF=183. F_{EC50}=209.9. F_{Hill}=2.339. Exact p values can be found in a supplemental file.

Table S5. Subunit specific effects of mutating residues contributing to conserved tripartite salt bridges at the ECD – TMD interfaces.

	Dose-response[a]				$[^{125}I]$-α-BTX[b,d]
Mutant	EC_{50} (μM)	Hill slope	n	Fold change	CPM_{mutant}/CPM_{WT}
WT	9.40 ± 1.82	1.78 ± 0.35	87	-	1.00 ± 0.24
<u>α subunit</u>					
αE45A	24.4 ± 6.1[e]	1.55 ± 0.10	8	2.62 loss	
αE45R	0.600 ± 0.316[e]	1.39 ± 0.21	8	15.6 gain	
αD138A	NE[c]			-	0.15 ± 0.05
αD138E	6.14 ± 0.69	1.93 ± 0.17	8	1.53 gain	
αD138R	NE[c]			-	0.10 ± 0.04
αE175A	49.7 ± 6.7[e]	1.69 ± 0.13	9	5.29 loss	
αE175R	17.3 ± 4.2[e]	1.58 ± 0.10	9	1.84 loss	
αR209A	NE[c]			-	0.04 ± 0.02
αR209Q	NE[c]			-	0.05 ± 0.03
αR209E	NE[c]			-	0.02 ± 0.02
<u>β subunit</u>					
βE45A	10.0 ± 1.4	1.68 ± 0.21	8	1.07 loss	
βE45R	10.3 ± 1.0	1.64 ± 0.16	8	1.09 loss	
βD138A	20.5 ± 2.6[e]	1.79 ± 0.16	8	2.18 gain	
βD138E	8.63 ± 1.74	1.66 ± 0.22	8	1.09 gain	
βD138R	9.98 ± 4.25	2.02 ± 0.75	8	1.06 gain	
βQ185A	10.6 ± 2.0	1.79 ± 0.23	9	1.12 loss	
βQ185R	13.2 ± 2.6	1.38 ± 0.23	9	1.41 loss	
βR215A	22.4 ± 6.1[e]	1.56 ± 0.58	8	2.39 loss	
βR215Q	16.0 ± 1.8[e]	1.63 ± 0.21	8	1.70 loss	
βR215E	18.2 ± 2.3[e]	1.70 ± 0.24	9	1.94 loss	
<u>δ subunit</u>					
δE47A	13.0 ± 2.1	2.32 ± 0.61	9	1.38 loss	
δE47R	15.7 ± 1.8[e]	1.90 ± 0.27	8	1.69 loss	
δD140A	2.58 ± 0.96[e]	1.66 ± 0.26	8	3.65 gain	
δD140E	9.55 ± 0.83	2.21 ± 0.29	8	1.02 gain	
δD140R	4.64 ± 1.12[e]	1.80 ± 0.66	8	2.20 gain	
δE189A	10.2 ± 1.1	1.80 ± 0.21	9	1.09 loss	
δE189R	34.5 ± 5.1[e]	1.86 ± 0.25	9	3.67 loss	
δR223A	7.02 ± 1.37	1.60 ± 0.26	8	1.33 gain	
δR223Q	7.36 ± 2.50	1.32 ± 0.48	8	1.28 gain	
δR223E	5.95 ± 2.09	1.99 ± 0.42	9	1.57 gain	
<u>γ subunit</u>					
γE45A	5.98 ± 0.74	1.95 ± 0.23	8	1.57 gain	
γE45R	12.3 ± 1.7	1.92 ± 0.26	8	1.31 loss	
γD138A	11.5 ± 2.3	1.90 ± 0.23	9	1.22 loss	
γD138E	12.0 ± 2.8	1.96 ± 0.40	8	1.27 loss	
γD138R	NE[c]		-		0.13 ± 0.02
γE183A	15.9 ± 3.3[e]	1.69 ± 0.15	8	1.69 loss	
γE183R	NE[c]		-	-	0.09 ± 0.03
γR217A	10.5 ± 1.5	2.05 ± 0.34	8	1.11 loss	
γR217Q	NE[c]		-	-	0.17 ± 0.05
γR217E	NE[c]		-	-	0.07 ± 0.03
Mock					0.07 ± 0.03

[a]Measurements performed 2-4 days after cRNA injection (V_{hold} = -60 mV). Error values represented as standard deviation
[b]Measurements performed 2 days after cRNA injection on a minimum of 10 oocyes. Error values represented as standard deviation
[c]No response (NE). No significant agonist induced current observed up to 4 day after cRNA injection
[d]None of the mutants tested were significantly ($p < 0.001$) more expressed than mock injected oocytes via one-way ANOVA followed by Dunnet's post hoc test
[e]$p < 0.001$ relative to WT via one-way ANOVA followed by Dunnet's post hoc test. DF=313. F_{EC50}=112.5. F_{Hill}=3.667. Exact p values can be found in a supplemental file.

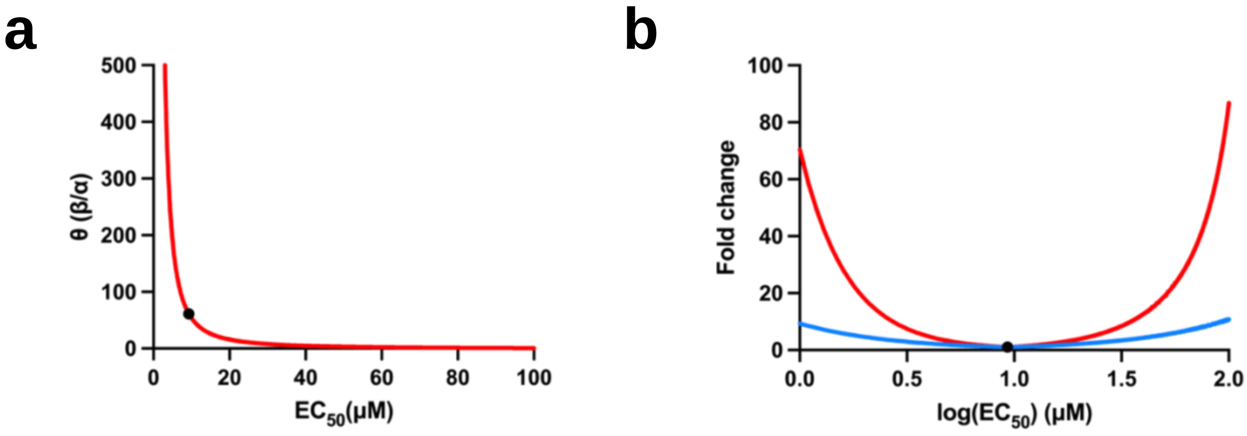

c

Mutant	*Torpedo* nAChR EC50 (μM)	*Torpedo* nAChR Fold change (EC50)	Human adult nAChR EC50 (μM)	Human adult nAChR Fold change (EC50)	Human adult nAChR θ (lit)[b]	Human adult nAChR Fold change (θ) (lit)[b]
WT	9.40	-	7.98	-	27.0	-
αE45R	0.600	0.063	1.40	0.176	4.10	6.59
αE45A	24.4	2.67	22.3	2.79	2.10	12.9
αE45K	NR[a]		3.63	0.457	0.224	121
αV46A	108	11.5	69.4	8.74	0.057	474
αP272G	27.1	2.88	37.9	4.77	0.170	159
αE45R+αR209Q	9.16	0.974	2.50	3.19	19.0	1.42
αE45R+αR209E	9.80	1.04	NR[a]			1.35
αE45A+αV46A	NR[a]		7.32	1.08	0.005	5,870
αE45A+αP272G	55.8	5.93	NR[a]		0.042	643
αV46A+αP272G	142	15.1	38.0	4.76	0.006	4,820
αE45A+V46A+αP272G	NR[a]		NR[a]		0.005	5,000

[a]No response (NR). No significant agonist induced current observed up to 4 days after cRNA injection.

[b]θ values from[1].

Figure S1. Relative sensitivities of EC_{50} versus θ to changes in channel function.

Measured changes in EC_{50} values relative to wild type for mutations in the muscle nAChR are typically of lesser magnitude than the changes observed in the di-liganded gating equilibrium constant, θ, for the same mutation. Measurements of EC_{50} are thus less sensitive to changes in channel gating. Such differences are expected, however, because EC_{50} values, which reflect both agonist binding (K_d) and channel gating (θ), are mathematically weighted towards the K_d, as illustrated by equation 3[2]:

$$EC_{50} = \frac{K_{d,R} * (1 + \sqrt{\theta + 2})}{\theta + 1} \qquad (3)$$

which assumes that gating only occurs from the di-liganded state:

$$R + 2L \overset{K_{d,R}}{\rightleftharpoons} RL_2 \overset{\theta}{\rightleftharpoons} OL_2$$

where R corresponds to the closed resting state, O the activated open state, and $K_{d,R}$ the resting state agonist (acetylcholine) affinity assuming both agonist sites are equivalent.

To explore the relationship between EC_{50} and θ, we first *estimated* the $K_{d,R}$ for both *Torpedo* (64.6 μM) and the human adult muscle (54.6 μM) nAChR using our measured EC_{50} values and a common θ value of 60,

which was determined elsewhere for the human adult muscle nAChR[3]. A) Assuming a constant $K_{d,R}$, we illustrate the non-linearity of the relationship between EC_{50} and θ by plotting the expected change in θ as a function of a change in EC_{50} value. The black dot represents WT values for the *Torpedo* nAChR. The non-linearity of the relationship is further illustrated in B), which compares how the fold change in θ relative to WT (red curve) manifests as a fold change in EC_{50} relative to WT (blue curve) as θ deviates from WT (black dot).

The two curves illustrate that the greater the functional effect of the mutant on channel gating (i.e., the greater the change in θ), the larger the discrepancy between the measured fold change in EC_{50} versus θ. For example, using these approximations, <u>2-, 5- and 10-fold changes</u> in EC_{50} in the Torpedo nAChR should correspond to roughly <u>3, 15 and 70-fold changes</u> in θ, respectively.

C) The table compares the experimentally measured EC_{50} values for select mutants in both the *Torpedo* and the adult human muscle nAChR, along with the fold change in each EC_{50} relative to WT (this study), to the changes in the di-liganded gating equilibrium constant, θ, calculated for each mutation in the adult human muscle nAChR from kinetic fitting of single channel measurements[1]. Considering the expected differences in the sensitivities of EC_{50} versus θ values to changes in channel function, the functional data obtained using both whole cell and single channel electrophysiological recordings present a consistent picture regarding the importance of residues at the intra-α subunit ECD-TMD domain interface. Despite structures showing that β1-β2 moves roughly orthogonally to M2-M3 upon agonist binding (Fig. 1b and Movie 1, panel a), the mutagenesis data reaffirms that the ECD triad plays an important role in channel gating.

Figure S2. Raw TEVC traces used to plot dose-response curves. Representative traces from select mutants are presented in the following seven pages as a change in current over time in response to increasing concentrations of ACh. Horizontal bars above each trace represent the time the oocyte was exposed to the indicated concentration of ACh.

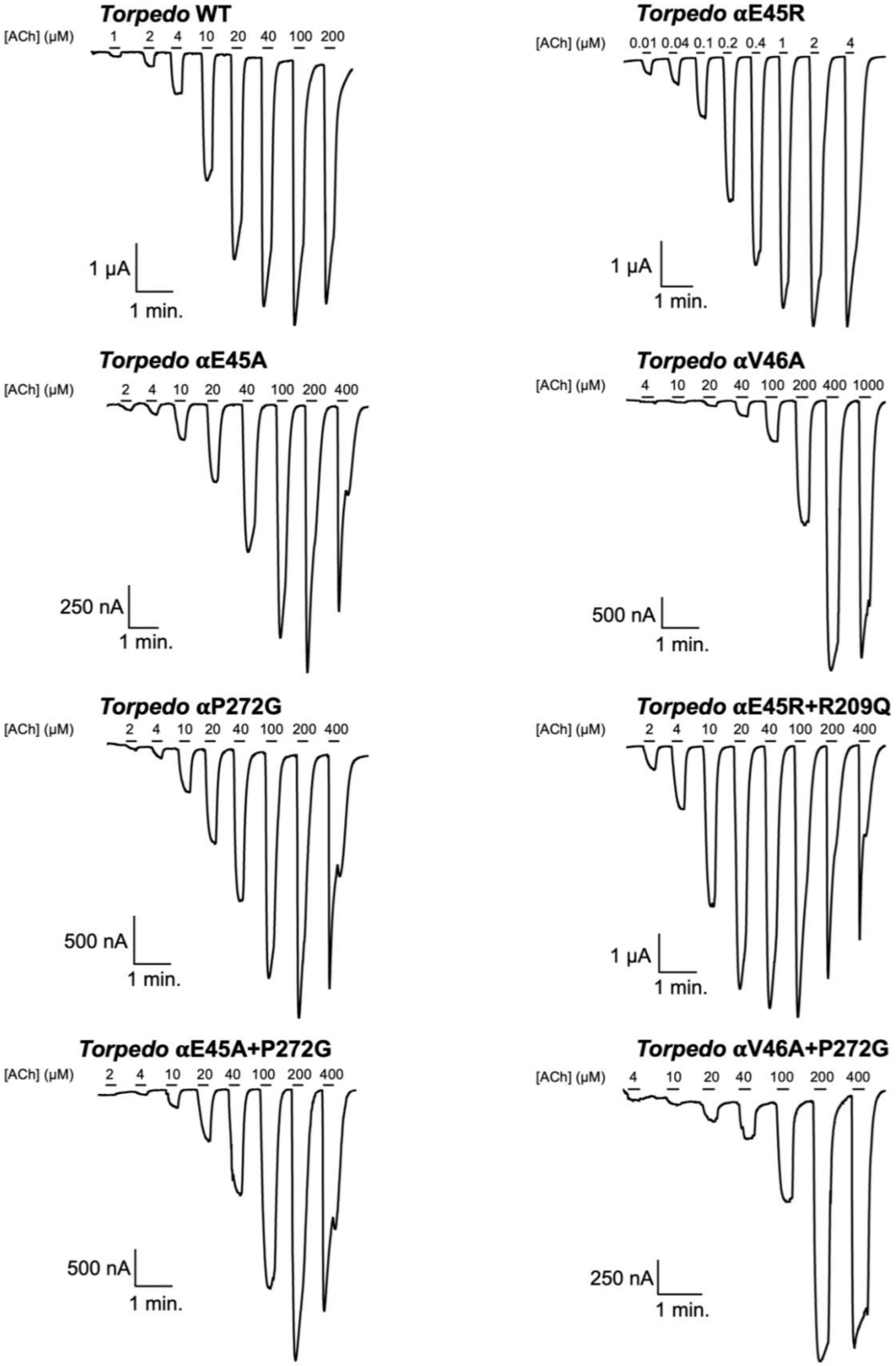
Torpedo WT
[ACh] (μM)
1 2 4 10 20 40 100 200
1 μA
1 min.
Torpedo αE45R
[ACh] (μM)
0.01 0.04 0.1 0.2 0.4 1 2 4
1 μA
1 min.
Torpedo αE45A
[ACh] (μM)
2 4 10 20 40 100 200 400
250 nA
1 min.
Torpedo αV46A
[ACh] (μM)
4 10 20 40 100 200 400 1000
500 nA
1 min.
Torpedo αP272G
[ACh] (μM)
2 4 10 20 40 100 200 400
500 nA
1 min.
Torpedo αE45R+R209Q
[ACh] (μM)
2 4 10 20 40 100 200 400
1 μA
1 min.
Torpedo αE45A+P272G
[ACh] (μM)
2 4 10 20 40 100 200 400
500 nA
1 min.
Torpedo αV46A+P272G
[ACh] (μM)
4 10 20 40 100 200 400
250 nA
1 min.

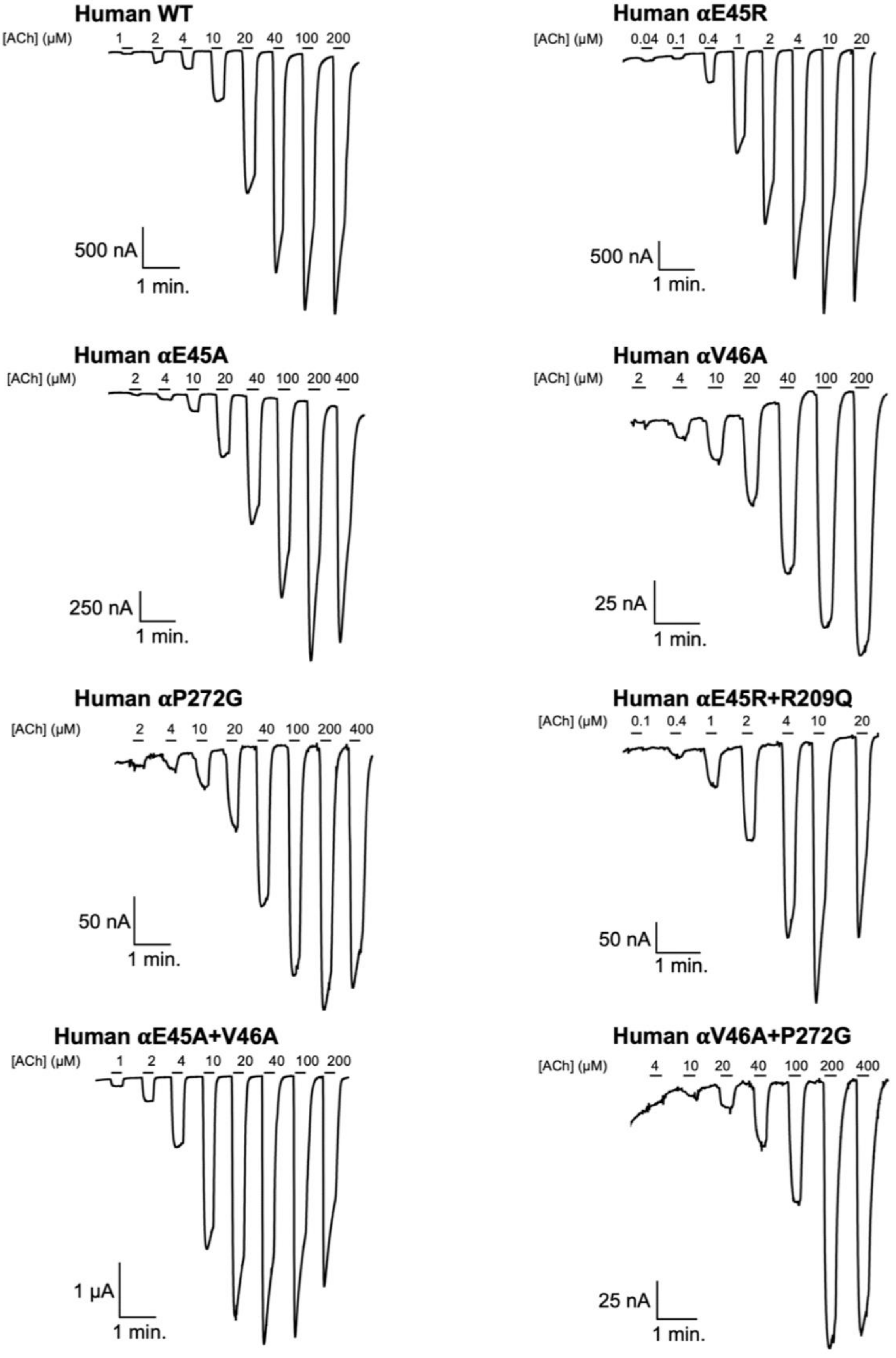
Human WT
[ACh] (μM) 1 2 4 10 20 40 100 200
500 nA
1 min.
Human αE45R
[ACh] (μM) 0.04 0.1 0.4 1 2 4 10 20
500 nA
1 min.
Human αE45A
[ACh] (μM) 2 4 10 20 40 100 200 400
250 nA
1 min.
Human αV46A
[ACh] (μM) 2 4 10 20 40 100 200
25 nA
1 min.
Human αP272G
[ACh] (μM) 2 4 10 20 40 100 200 400
50 nA
1 min.
Human αE45R+R209Q
[ACh] (μM) 0.1 0.4 1 2 4 10 20
50 nA
1 min.
Human αE45A+V46A
[ACh] (μM) 1 2 4 10 20 40 100 200
1 μA
1 min.
Human αV46A+P272G
[ACh] (μM) 4 10 20 40 100 200 400
25 nA
1 min.

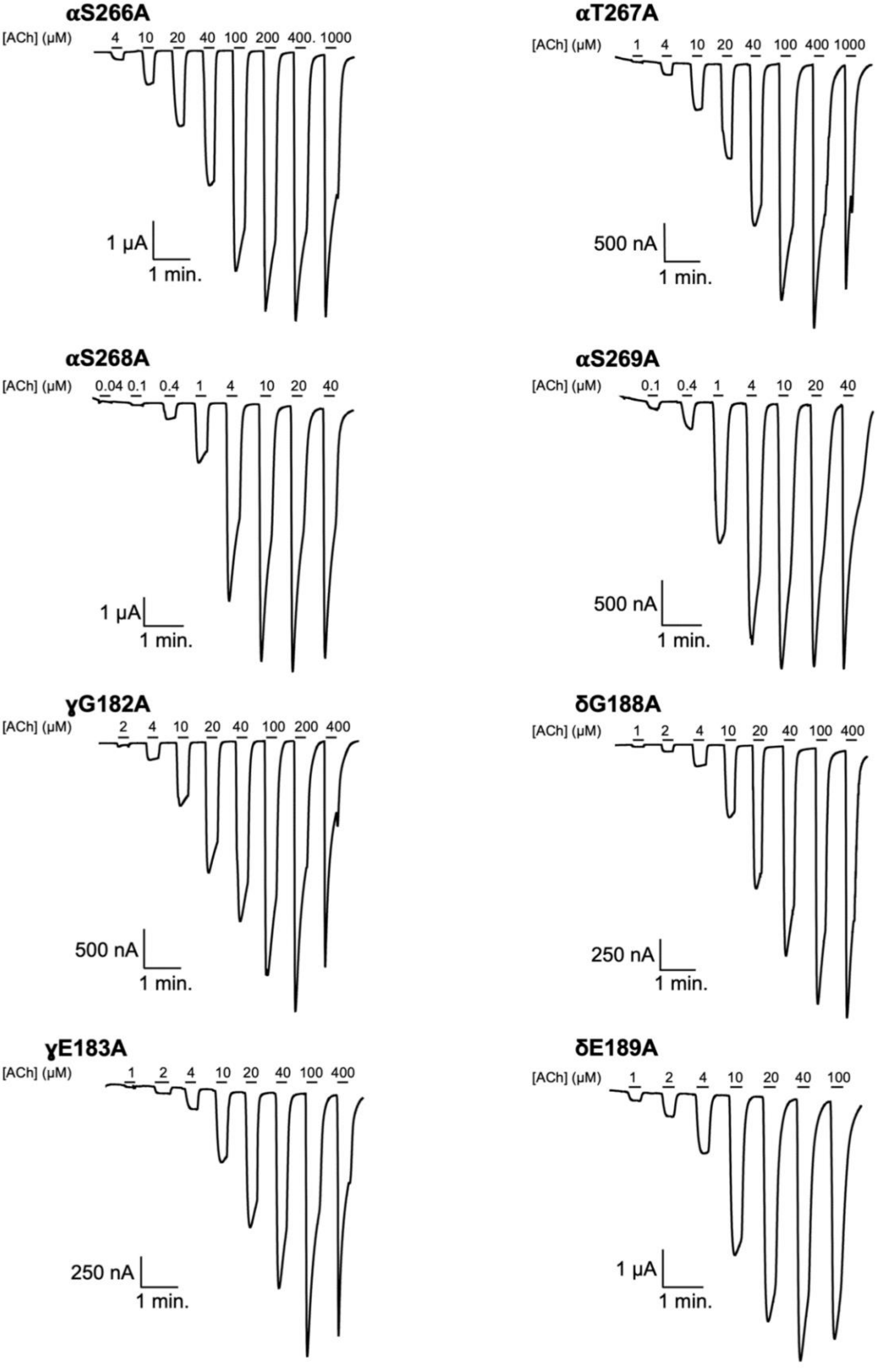
αS266A
[ACh] (μM) 4 10 20 40 100 200 400. 1000
1 μA
1 min.
αT267A
[ACh] (μM) 1 4 10 20 40 100 400 1000
500 nA
1 min.
αS268A
[ACh] (μM) 0.04 0.1 0.4 1 4 10 20 40
1 μA
1 min.
αS269A
[ACh] (μM) 0.1 0.4 1 4 10 20 40
500 nA
1 min.
γG182A
[ACh] (μM) 2 4 10 20 40 100 200 400
500 nA
1 min.
δG188A
[ACh] (μM) 1 2 4 10 20 40 100 400
250 nA
1 min.
γE183A
[ACh] (μM) 1 2 4 10 20 40 100 400
250 nA
1 min.
δE189A
[ACh] (μM) 1 2 4 10 20 40 100
1 μA
1 min.

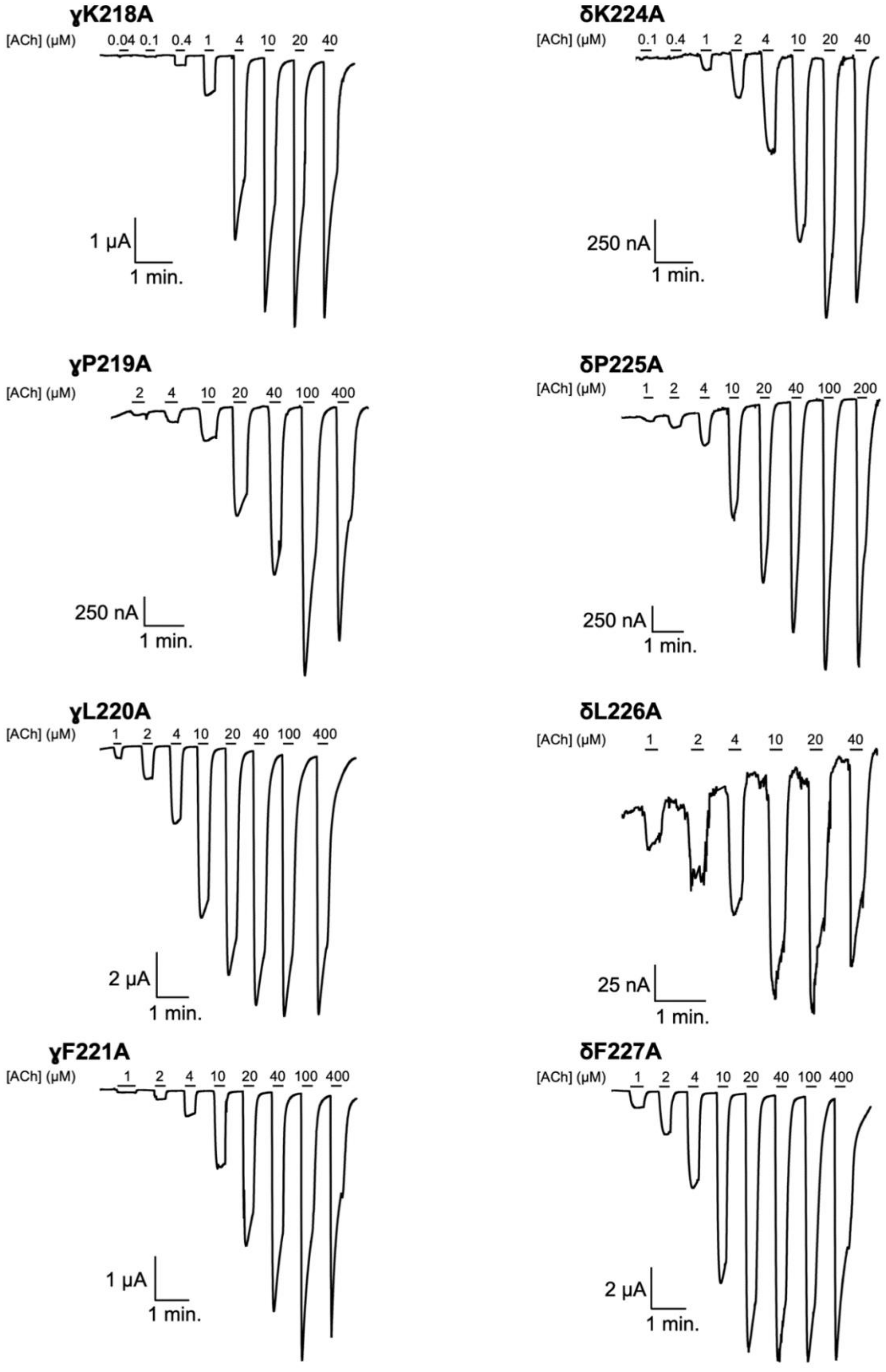
γK218A
[ACh] (μM)
0.04 0.1 0.4 1 4 10 20 40
1 μA
1 min.
δK224A
[ACh] (μM)
0.1 0.4 1 2 4 10 20 40
250 nA
1 min.
γP219A
[ACh] (μM)
2 4 10 20 40 100 400
250 nA
1 min.
δP225A
[ACh] (μM)
1 2 4 10 20 40 100 200
250 nA
1 min.
γL220A
[ACh] (μM)
1 2 4 10 20 40 100 400
2 μA
1 min.
δL226A
[ACh] (μM)
1 2 4 10 20 40
25 nA
1 min.
γF221A
[ACh] (μM)
1 2 4 10 20 40 100 400
1 μA
1 min.
δF227A
[ACh] (μM)
1 2 4 10 20 40 100 400
2 μA
1 min.

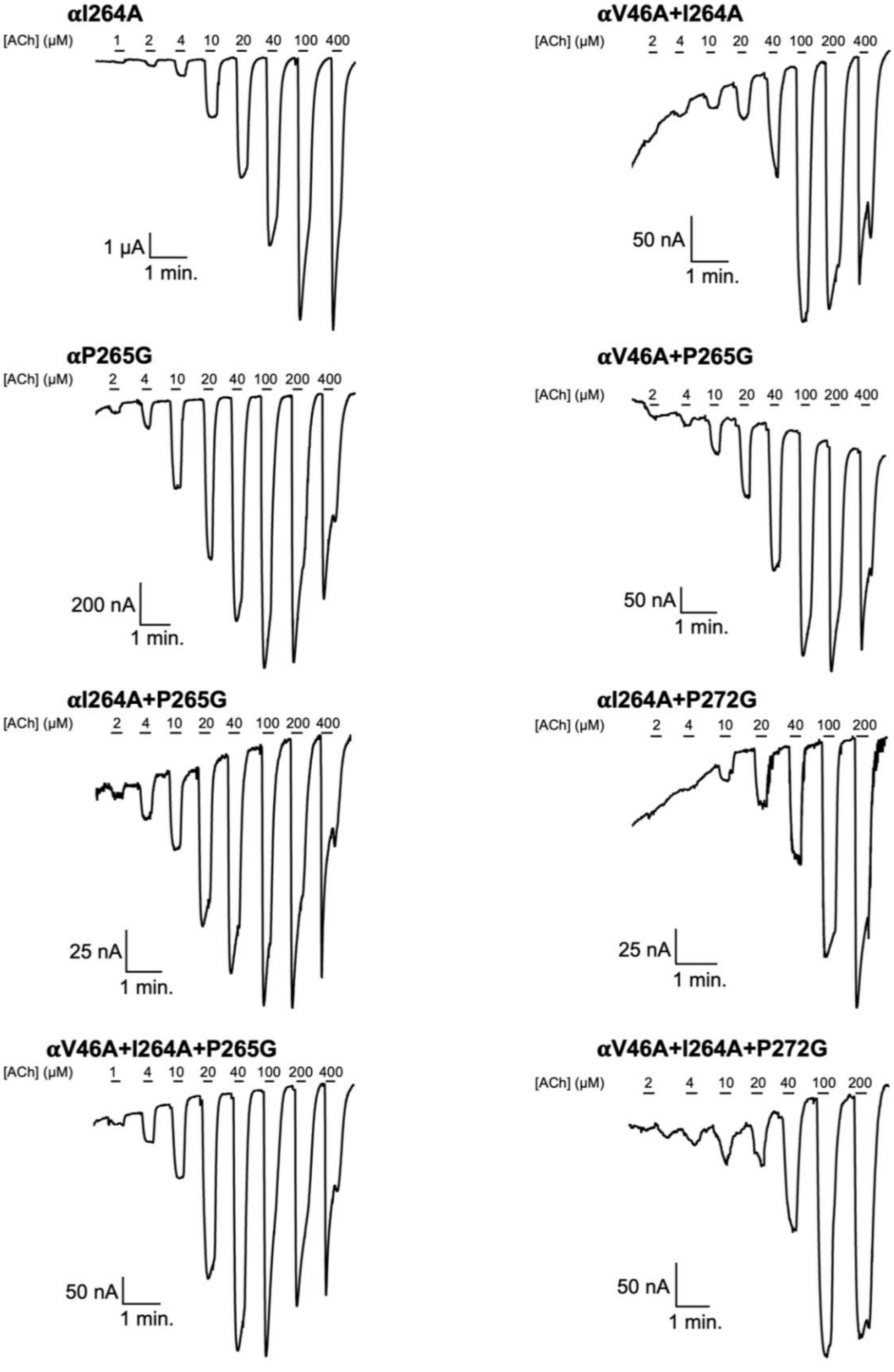
αI264A
[ACh] (μM) 1 2 4 10 20 40 100 400
1 μA
1 min.
αV46A+I264A
[ACh] (μM) 2 4 10 20 40 100 200 400
50 nA
1 min.
αP265G
[ACh] (μM) 2 4 10 20 40 100 200 400
200 nA
1 min.
αV46A+P265G
[ACh] (μM) 2 4 10 20 40 100 200 400
50 nA
1 min.
αI264A+P265G
[ACh] (μM) 2 4 10 20 40 100 200 400
25 nA
1 min.
αI264A+P272G
[ACh] (μM) 2 4 10 20 40 100 200
25 nA
1 min.
αV46A+I264A+P265G
[ACh] (μM) 1 4 10 20 40 100 200 400
50 nA
1 min.
αV46A+I264A+P272G
[ACh] (μM) 2 4 10 20 40 100 200
50 nA
1 min.

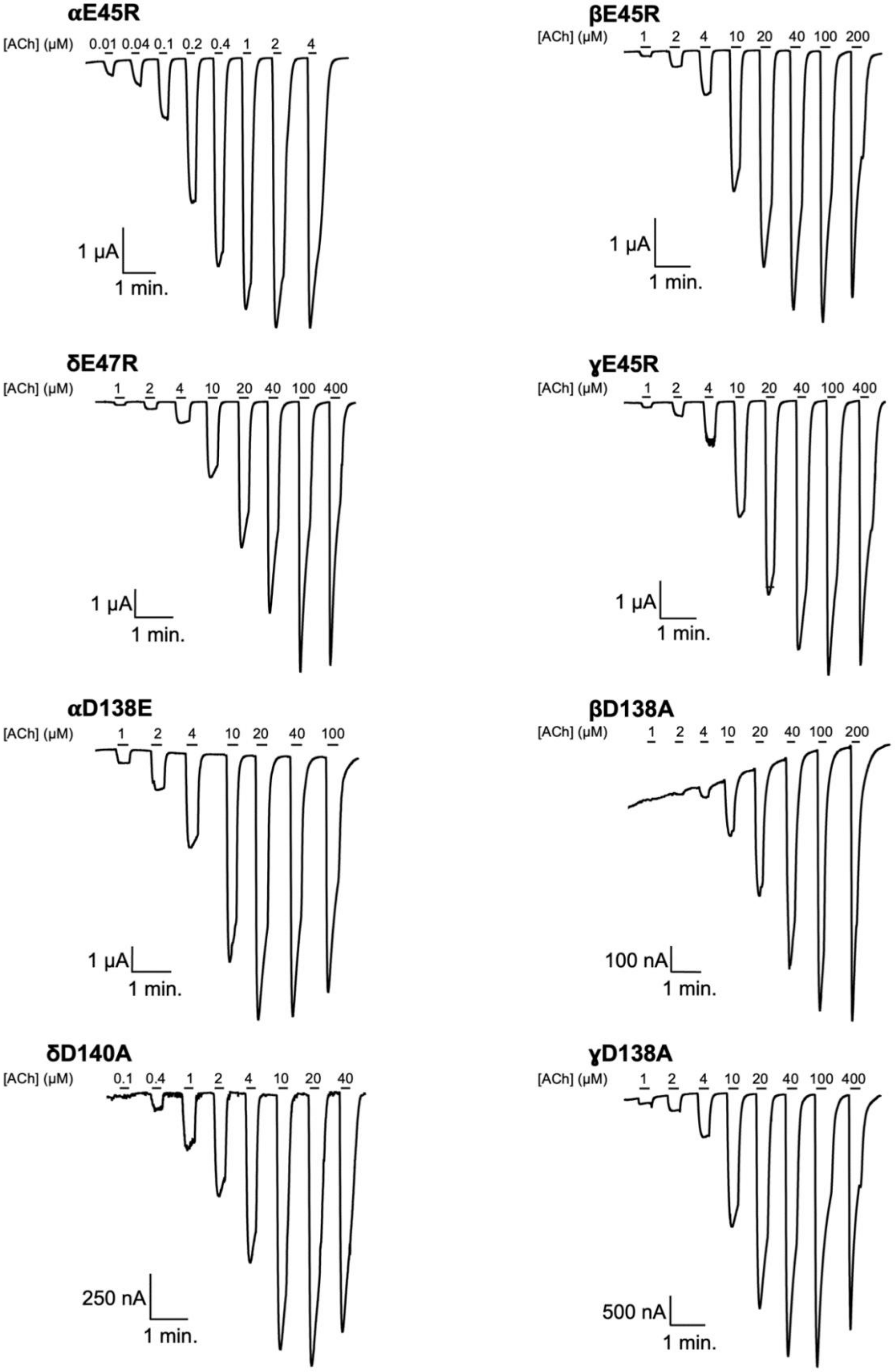
αE45R
[ACh] (μM) 0.01 0.04 0.1 0.2 0.4 1 2 4
1 μA
1 min.
βE45R
[ACh] (μM) 1 2 4 10 20 40 100 200
1 μA
1 min.
δE47R
[ACh] (μM) 1 2 4 10 20 40 100 400
1 μA
1 min.
γE45R
[ACh] (μM) 1 2 4 10 20 40 100 400
1 μA
1 min.
αD138E
[ACh] (μM) 1 2 4 10 20 40 100
1 μA
1 min.
βD138A
[ACh] (μM) 1 2 4 10 20 40 100 200
100 nA
1 min.
δD140A
[ACh] (μM) 0.1 0.4 1 2 4 10 20 40
250 nA
1 min.
γD138A
[ACh] (μM) 1 2 4 10 20 40 100 400
500 nA
1 min.

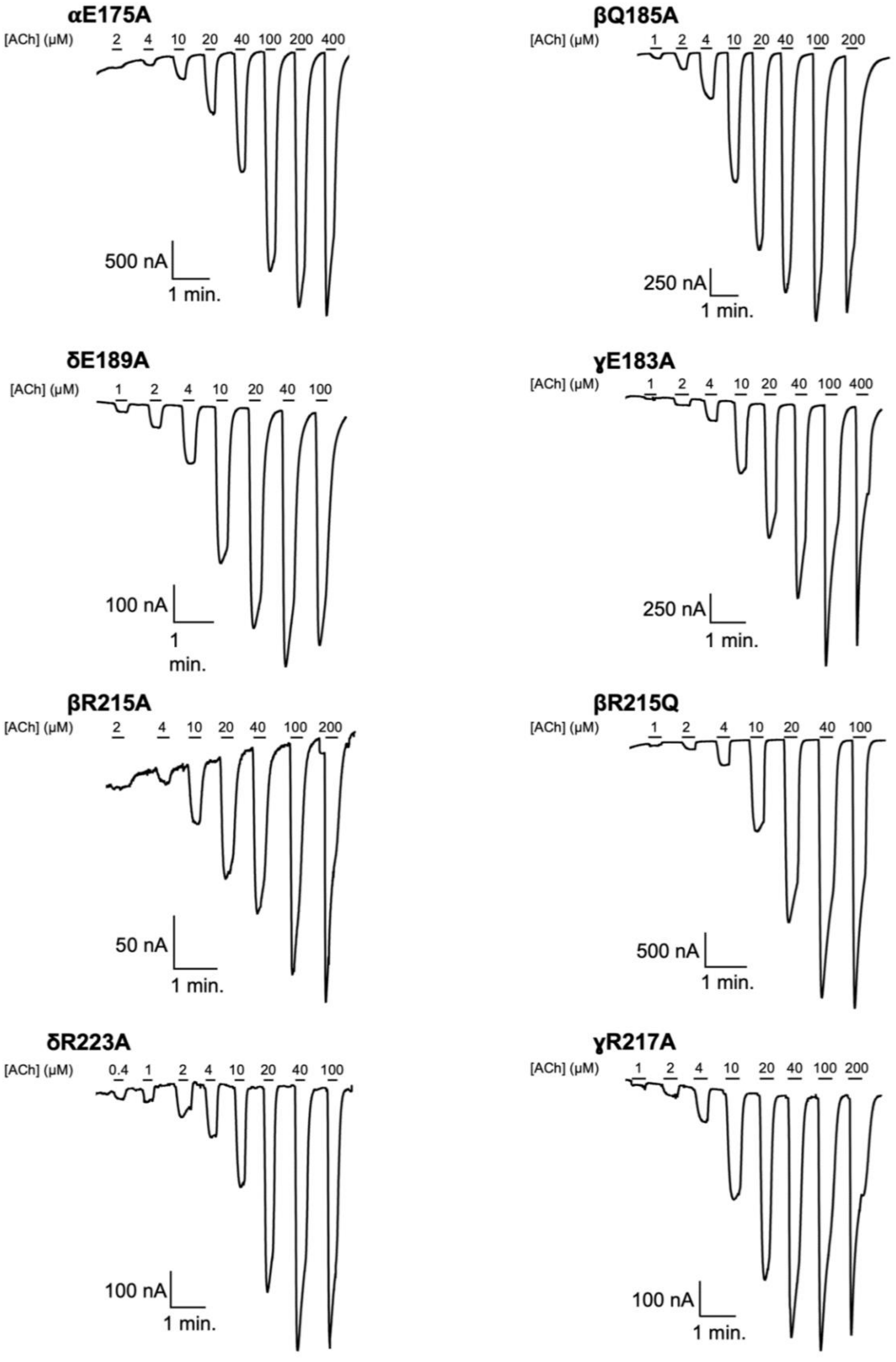
αE175A
[ACh] (μM)
2 4 10 20 40 100 200 400
500 nA
1 min.
βQ185A
[ACh] (μM)
1 2 4 10 20 40 100 200
250 nA
1 min.
δE189A
[ACh] (μM)
1 2 4 10 20 40 100
100 nA
1 min.
ɣE183A
[ACh] (μM)
1 2 4 10 20 40 100 400
250 nA
1 min.
βR215A
[ACh] (μM)
2 4 10 20 40 100 200
50 nA
1 min.
βR215Q
[ACh] (μM)
1 2 4 10 20 40 100
500 nA
1 min.
δR223A
[ACh] (μM)
0.4 1 2 4 10 20 40 100
100 nA
1 min.
ɣR217A
[ACh] (μM)
1 2 4 10 20 40 100 200
100 nA
1 min.

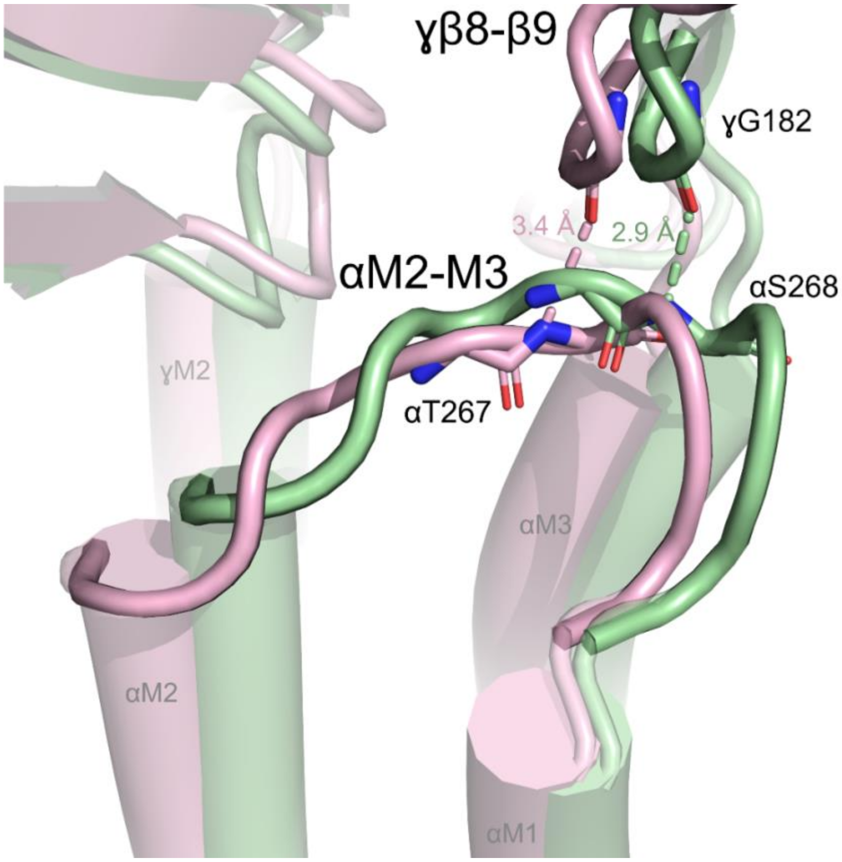

	Dose-response[a]						Fold-change
	WT			αS268P			EC_{50}(WT)
Mutant	EC_{50} (μM)	Hill slope	n	EC_{50} (μM)	Hill slope	n	EC_{50}(S268P)
WT	9.40 ± 1.82	1.78 ± 0.35	87	9.67 ± 1.69	1.81 ± 0.41	8	0.97
γG182A	23.4 ± 2.8[b]	1.53 ± 0.26	8	7.81 ± 1.71	1.67 ± 0.18	9	3.00
δG188A	19.3 ± 3.5[b]	1.59 ± 0.20	8	11.5 ± 0.7	1.62 ± 0.13	8	1.68
γG182A+δG188A	31.0 ± 2.1[b]	1.68 ± 0.30	9	6.50 ± 2.47	1.63 ± 0.55	8	4.77
αS266A+αT267A	31.4 ± 3.3[b]	1.99 ± 0.12	8	13.9 ± 1.3[c]	1.65 ± 0.19	10	2.26
αS266A+αT267A+γG182A	33.0 ± 5.6[b]	1.87 ± 0.26	9	8.43 ± 2.67	2.14 ± 1.0	8	3.91
αS266A+αT267A+δG188A	37.2 ± 1.8[b]	1.82 ± 0.31	9	16.1 ± 1.9[c]	1.66 ± 0.45	8	2.31
αS266A+αT267A+γG182A+δG188A	31.5 ± 4.6[b]	2.13 ± 0.32	9	NR[d]			-

[a]Measurements performed 2-4 days after cRNA injection (V_{hold} = -60 mV). Error values represented as standard deviation

[b]$p < 0.001$ relative to WT via one-way ANOVA followed by Dunnet's post hoc test. DF=139. F_{EC50}=332.7. F_{Hill}=3.011. Exact p values can be found in a supplemental file.

[c]$p < 0.001$ relative to αS268P via one-way ANOVA followed by Dunnet's post hoc test. DF=52. F_{EC50}=20.84. F_{Hill}=1.194. Exact p values can be found in a supplemental file.

[d]No response (NR). No significant agonist induced current observed up to 4 day after cRNA injection

Figure S3. The interdependence between γGly182/δGly188 and the αγ/αδ M2-M3 loops is mediated by a backbone hydrogen bond with αSer268. Apo (pink) and agonist-bound (green) states of the nAChR are shown in panel a with a dashed line highlighting the backbone hydrogen bond between γGly182 and αSer268. The αS268P mutant has almost no effect on channel function alone but eliminates the effect of the γG182A/δG188A double mutant.

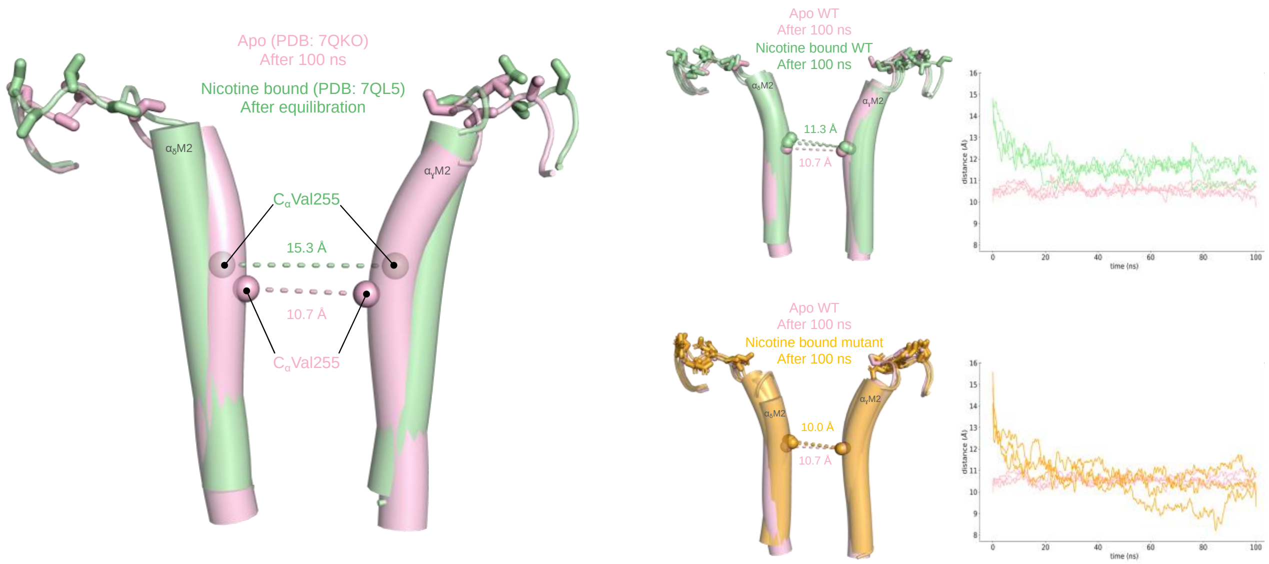

Figure S4. The quadruple Asn αM2-M3 mutant stabilizes the resting state. The M2 α-helices and M2-M3 loops of both α subunits are shown on the left for the WT apo state after 100 ns of simulation (pink) and the WT nicotine state after minimization but prior to the simulation (green). Residues in the M2-M3 loop are shown as sticks and the α carbons of αVal255 (V13') are shown as spheres with the distance between the two depicted by dashed lines to highlight the difference in pore diameter. Three repeat all atom simulations of the apo nAChR (top right, green traces) show that the pore is stably closed. In contrast, unless the diffuse density observed in the pore is modeled with bound lipids (see[4]), the open pore of the nicotine bound structure closes within ~40 ns to a conformation with a diameter slightly larger than the apo state (top right, orange traces). On the bottom right, the pore diameter of the quadruple Asn mutant (orange) after 100 ns simulation is compared to the apo WT (green) after 100 ns simulation. The average distance between αV13' residues from the three repeats show that the quadruple Asn mutation in M2-M3 accelerates the collapse of the pore all the way back to the apo WT state.

Supplemental Discussion of the conformation adopted by the ECD of each subunit in apo and agonist bound forms of the *Torpedo* nAChR.

To assess whether the local conformational asymmetry observed at the interface between the β1-β2 and M2-M3 loops in different subunits extends to the entire ECD of each subunit, as originally proposed by Unwin et al.[5], we used several parameters including those developed by Lev et al.[6] and Calimet et al.[7].

To assess the capping of loop C, we first analyzed the apparent counterclockwise rotation of each subunit's ECD that occurs during activation. To capture these changes, we aligned the TMDs of apo and bound states and calculated the ECD twist angle using the metric documented in ref 6. Although GLIC undergoes an ~2° decrease in twist angle between the closed and open states, there is essentially no change in twist angle for the α subunits of the nAChR (Figure S5, panel B). The non-α subunits also have little change in twist angle but appear to increase rather than decrease during gating. It appears that there are differences in the twisting motions between homomeric and heteromeric pLGICs.

We quantified the ECD motions by measuring both the solvent accessible surface area (SASA) at the different ECD-ECD interfaces and the binding site contraction[7]. The SASA of the subunit-subunit interfaces in the ECD decreased in all subunits (Fig. S5, panel C), but the largest decreases occurred in both α subunits primarily due to a larger binding site contraction (Fig. S5, panel D). Neither metric seem to provide any evidence for pre-activation elsewhere in the ECD of the non-α subunits. The loop C capping of the non-α subunits, which should be captured in the binding site contraction metric, did not provide evidence for pre-existence in either apo or bound states. This is likely because the non-α subunit C loops adopt unique conformations that are morphologically dissimilar to the α subunits (Fig. S5, panel A) and are therefor difficult to directly compare.

We next used three previously defined metrics of activation, upper and lower ECD spreading and β-expansion, to assess whether the non-α subunits adopt a pre-active conformation. ECD spreading was calculated as the distance between the center of mass (COM) <u>of the entire ECD</u> at both the extracellular (upper) and TMD adjacent (lower) ends, to the COM <u>of each subunit</u> at the upper and lower ends. To find the COMs at the upper and lower ends of the entire ECD and the individual subunit ECD, an ellipsoid around the targets were created using the "measure inertia" command on ChimeraX using just the Cα's of conserved β-strands in each subunit. The COM of the ellipsoid, along with the vector of the longest principal axis (corresponding to the height axis of the domain), were used to find COMs at two z positions at the top and bottom of each ECD (see panel A of Fig. S6). The distance between the full ECD COM and the subunit COM in both apo and bound states is defined as the ECD spread (ref. 6) and is plotted on the right section of panel A of Fig. S6. We found that there is a small inward motion of the upper ECD from each subunit, with no obvious change between a and non-a subunits. At the lower ECD, there does seem to be a subtle difference in spreading in a versus non-a subunits with a larger spreading occurring in the non-a subunits. This is a product of the scissor-like motion that occurs during activation in the α subunits that is described in Zarkadas et al.[4]. The magnitudes of the ECD spreads do not provide evidence for a pre-active ECD conformation of the non-α subunits.

We also used β-expansion, a parameter used for both GLIC[6] and the homomeric ρ1 $GABA_AR$[8] to measure activation. We used the same protocol as previously described taking the COM of the Cα's from two five residues stretches (one in the β1-β2 loop and one in the β10-M1 linker) and measuring the distance in both apo and agonist bound state for each subunit (see panel B of the Fig. S6). The expansion was originally described from the open to closed state of GLIC and thus is a contraction in the activation transition. We also see a subtle contraction in the α subunits, but the non-α subunits instead expand. It is notable however

that for both homomeric receptors, the magnitude of the contraction are ~2Å , while here the magnitudes of the α subunit contractions are <0.2Å and the non-α subunit expansions are <0.8Å. Furthermore, neither the contractions in the α subunits nor the expansions in non-α subunits suggest a pre-activate like conformation of the non-α subunits in the apo state.

Finally, we examined the changes in tilt angles that occur in each subunit ECD upon agonist binding. This was done in two ways: first by assessing how much each ECD changes its tilt angle along the z plane (relative to the pore) during activation and second by determining the relative change in tilt angle between the ECD and TMD in each subunit during activation. The former was measured by calculating the change in angle between the vector derived from longest principal axis used to create the ellipsoids around each ECD and the z-axis (or pore axis). During activation, each subunit (other than the δ subunit) undergoes a straightening motion that decreases the angle relative to the pore axis (panels A and B in Fig. S7). This motion is conserved, and of a similar magnitude, in α and non-α subunits leaving no evidence for pre-activation of non-α subunits. It is notable however that while the non-α subunits seem to pivot with their membrane juxtaposed ends staying in the same position, the α subunits seem to translate toward the pore axis while also tilting (Fig. S7 panel A). Because each subunit was aligned by their TMD, the relative translation of the α subunit ECD suggests the angle between ECD and TMD in the α subunits would differ from the non-α subunits. To measure this, we calculated the change in angle between the vectors of the longest principal axis of ellipsoids around the ECD and TMD of the same subunit for both apo and bound states. Panel C shows that the interdomain tilt angle in the non-α subunits increase during activation but decreases in the α subunits due to their scissor like interdomain motions.

Altogether, we were not able to identify any evidence for the pre-active like conformation of the ECD – TMD interfaces of the non-α subunit extending more globally to the remainder of the ECD.

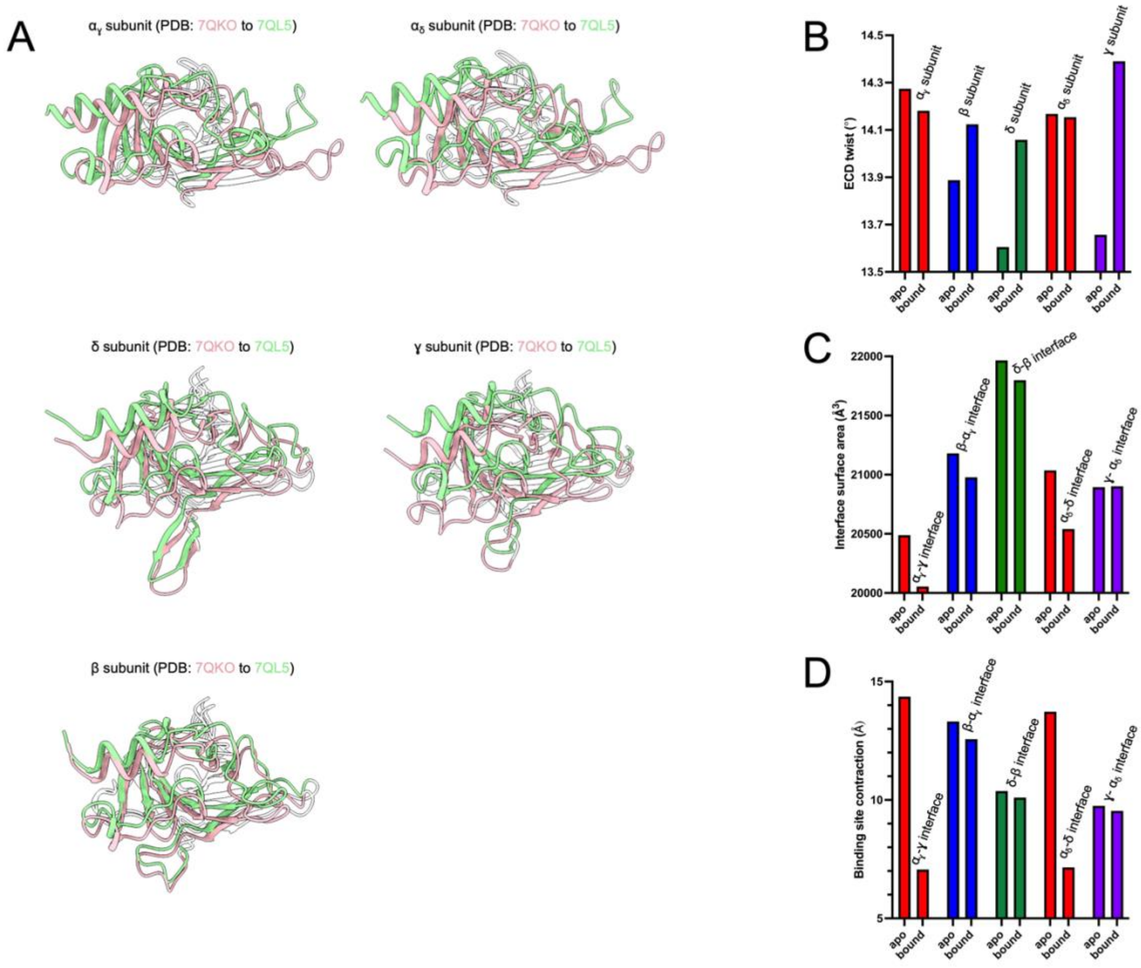

Figure S5. ECD twist does not provide evidence for pre-activation. Panel A shows top-down views of each subunit in the nAChR aligned by their TMDs in apo (pink) and agonist bound (green) states to show the relative motion of the ECD. Panel B shows change in ECD twist angle as defined in ref. 6. Panel C shows changes in solvent accessible surface area at each ECD – ECD interface. Panel D shows binding site contraction at each subunit interface as defined in ref. 7.

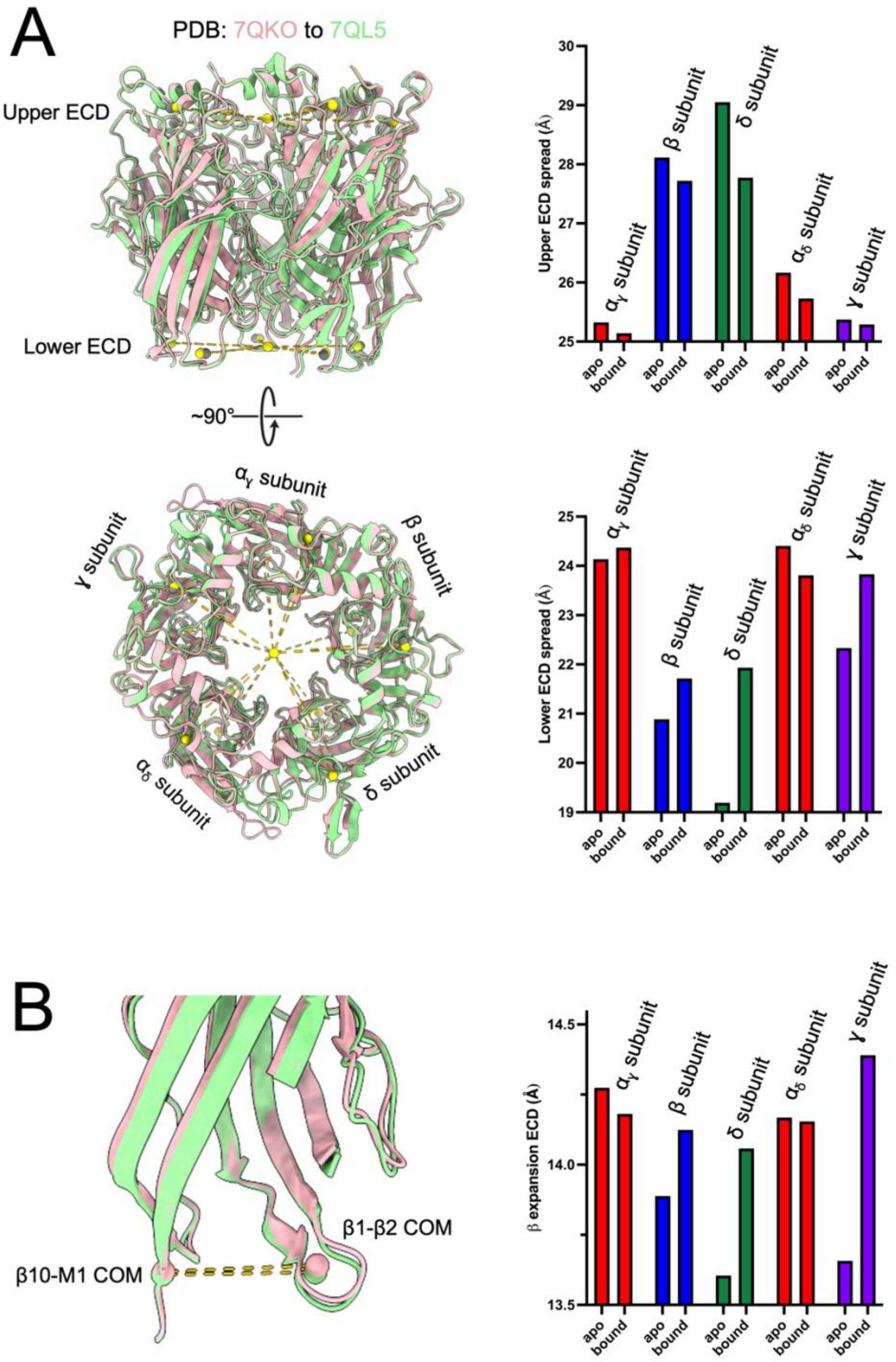

Figure S6. ECD spreading and β-expansion. Upper and lower ECD spreading (defined in ref. 6) is quantified for each subunit in panel A. The left shows the ECD of the apo (pink) and agonist bound (green) states aligned by their entire TMDs. Center of mass (COM) for the ECD of each subunit as well as the COM for the entire ECD are shown as spheres and the distances are depicted as dashed lines and plotted in the graph on the right. Panel B shows β-expansion in each subunit (also defined in ref. 6) with the minimal changes shown in both the models (left) and the graph (right).

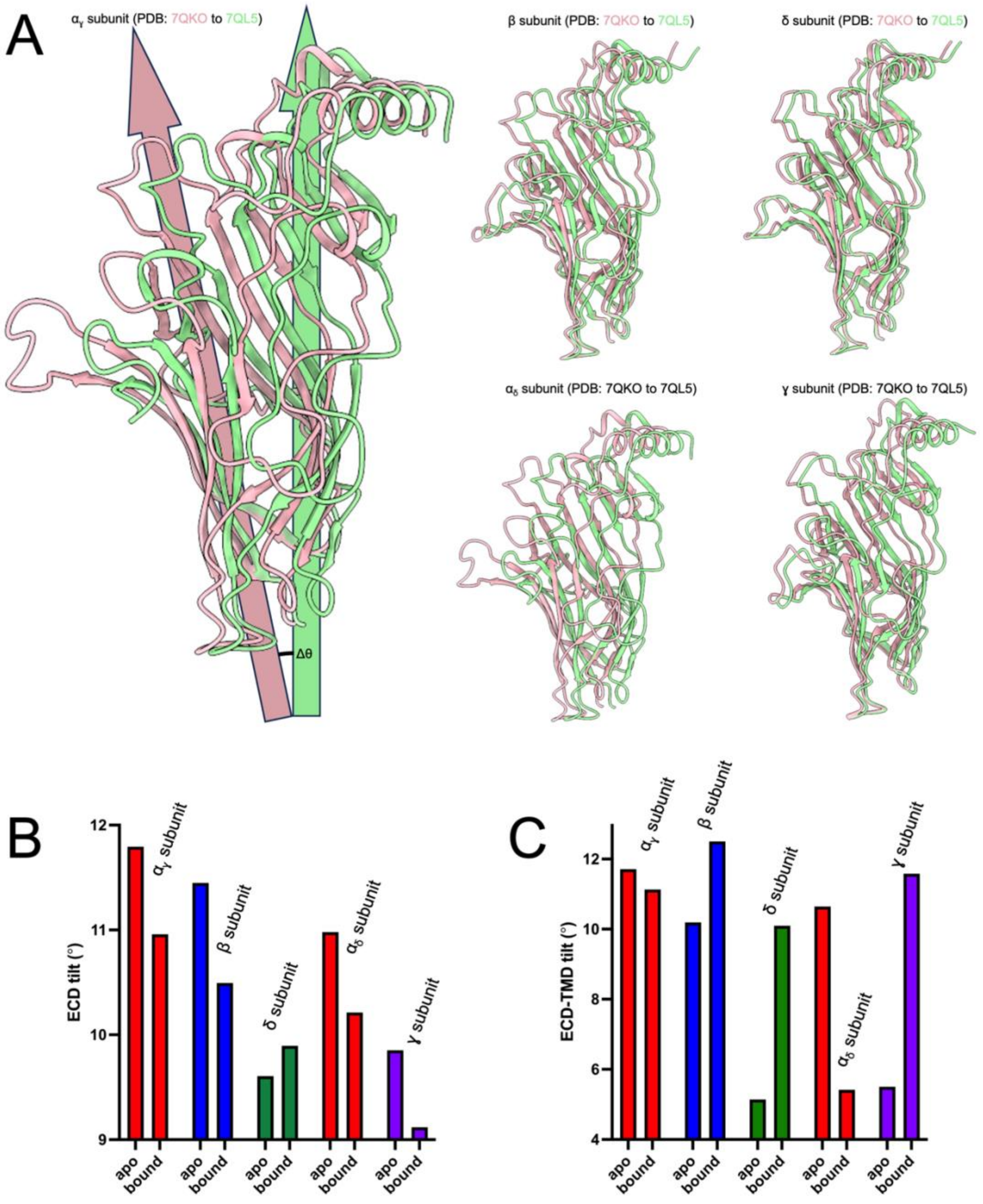

Figure S7. ECD-tilt angles. A COM and vector corresponding to the principal axis through the COM were defined for each subunit ECD using only the Cα carbons from the conserved β stands in each subunit. The ECD tilt was defined as the angle between the principal ECD axis and the pore axis for each subunit. A COM and vector corresponding to the principal axis for each TMD was then defined and the angle between the vectors for the principal ECD and TMD axis was defined as the ECD-TMD tilt.

References

1. Lee, W. Y. & Sine, S. M. Principal pathway coupling agonist binding to channel gating in nicotinic receptors. *Nature* **438**, 243–247 (2005).
2. Cecchini, M. & Changeux, J.-P. Nicotinic receptors: From protein allostery to computational neuropharmacology. *Mol. Aspects Med.* **84**, 101044 (2022).
3. Mukhtasimova, N., daCosta, C. J. B. & Sine, S. M. Improved resolution of single channel dwell times reveals mechanisms of binding, priming, and gating in muscle AChR. *J. Gen. Physiol.* **148**, 43–63 (2016).
4. Zarkadas, E. *et al.* Conformational transitions and ligand-binding to a muscle-type nicotinic acetylcholine receptor. *Neuron* **110**, 1358-1370.e5 (2022).
5. Unwin, N., Miyazawa, A., Li, J. & Fujiyoshi, Y. Activation of the nicotinic acetylcholine receptor involves a switch in conformation of the alpha subunits. *J. Mol. Biol.* **319**, 1165–1176 (2002).
6. Lev, B. *et al.* String method solution of the gating pathways for a pentameric ligand-gated ion channel. *Proc. Natl. Acad. Sci. U. S. A.* **114**, E4158–E4167 (2017).
7. Calimet, N. *et al.* A gating mechanism of pentameric ligand-gated ion channels. *Proc. Natl. Acad. Sci. U. S. A.* **110**, E3987-96 (2013).
8. Cowgill, J. *et al.* Structure and dynamics of differential ligand binding in the human ρ-type GABAA receptor. *bioRxiv* 2023.06.16.545288 (2023) doi:10.1101/2023.06.16.545288.
