# Supplementary material for: A release of local subunit conformational heterogeneity underlies gating in a muscle nicotinic acetylcholine receptor
Source: Nat Commun. 2024 Feb 27;15:1803. doi: 10.1038/s41467-024-46028-x (PMC10899235; doi:10.1038/s41467-024-46028-x)
Supplement: Supplementary file 3 — Description of Additional Supplementary Files [file 41467_2024_46028_MOESM3_ESM.pdf]

File Name: Movie 1

Description: Motions at the intra- and inter-subunit ECD – TMD interfaces during activation. The principal α_γ (salmon) and complementary γ (light blue) subunits are shown from two orthogonal views transitioning from apo to agonist bound states. The agonist is shown in cyan sticks. (a) Loop C capping tilts the α subunit ECD moving the αβ1-β2 loop away from the complementary subunit and towards the pore while the αM2-M3 loop moves orthogonally away from the pore. (b) The capping of loop F in the complementary subunits pivots the β8-β9 loops and the β10-M1 linkers away from the channel pore. These motions are correlated with the movement of the αM2-M3 loops away from pore.

File Name: Movie 2

Description: Asymmetric to symmetric transitions at the ECD – TMD interface. The top panel shows the relative movements of the Val46 and Pro265 residues in the α_γ and each of the non-α subunits when going from the apo to the agonist bound state. The bottom panel shows each of the three non-α subunits aligned to the α_γ subunit by their M2-M3 loops. The structures start in the apo state where they are asymmetric and transition into a symmetric agonist bound state.

File Name: Movie 3

Description: Tripartite salt bridges undergo larger displacements during the apo to agonist bound transition in α vs non-α subunits. Side (top) and top down (bottom) views of the tripartite salt bridges transitioning from apo to agonist bound conformations are shown for each subunit. The tripartite salt bridges in the two α subunits undergo large displacements relative to the TMD during the transition while the non-α subunits do not.

File Name: Movie 4

Description: Comparison of the Val – Pro motion in the *Torpedo* α subunit with other heteromeric and homomeric pLGICs. Apo to active morphs of each subunit were created once each subunit was aligned by their M2-M3 loop to view the relative motion of the Val46 equivalent. The α_γ subunit of the *Torpedo* nAChR is shown in orange and motion played in concert with three heteromeric pLGIC subunits (from left to right: the *Torpedo* nAChR β subunit, the principal agonist binding $GABA_A$R β2 subunit, and the complementary $GABA_A$R α1 subunit) and three homomeric pLGIC subunits (from left to right: the α7 nAChR, the $5HT_{3A}$R, and the α1 GlyR).

File Name: Movie 5

Description: Top view of the Val – Pro motion in the various pLGICs. The same morphs depicted in Movie 4 are shown from a top view looking from the ECD down toward the M2-M3 loop. A principle (left) and non-principle (right) agonist binding subunit from the heteromeric *Torpedo* nAChR (top) and synaptic $GABA_A$R (bottom) are shown in the top box. Homomeric α7 nAChR (left) and α1 GlyR (right) subunits are shown in the bottom box.
