# Supplementary material for: A release of local subunit conformational heterogeneity underlies gating in a muscle nicotinic acetylcholine receptor
Source: Nat Commun. 2024 Feb 27;15:1803. doi: 10.1038/s41467-024-46028-x (PMC10899235; doi:10.1038/s41467-024-46028-x)
Supplement: Supplementary file 9 — Reporting Summary [file 41467_2024_46028_MOESM9_ESM.pdf]

nature portfolio

Corresponding author(s): John E. Baenziger

Last updated by author(s): Jun 7, 2023

# Reporting Summary

Nature Portfolio wishes to improve the reproducibility of the work that we publish. This form provides structure for consistency and transparency in reporting. For further information on Nature Portfolio policies, see our Editorial Policies and the Editorial Policy Checklist.

## Statistics

For all statistical analyses, confirm that the following items are present in the figure legend, table legend, main text, or Methods section.

| n/a | Confirmed | |
|---|---|---|
| ☐ | ☒ | The exact sample size (*n*) for each experimental group/condition, given as a discrete number and unit of measurement |
| ☐ | ☒ | A statement on whether measurements were taken from distinct samples or whether the same sample was measured repeatedly |
| ☐ | ☒ | The statistical test(s) used AND whether they are one- or two-sided<br>*Only common tests should be described solely by name; describe more complex techniques in the Methods section.* |
| ☒ | ☐ | A description of all covariates tested |
| ☒ | ☐ | A description of any assumptions or corrections, such as tests of normality and adjustment for multiple comparisons |
| ☐ | ☒ | A full description of the statistical parameters including central tendency (e.g. means) or other basic estimates (e.g. regression coefficient) AND variation (e.g. standard deviation) or associated estimates of uncertainty (e.g. confidence intervals) |
| ☐ | ☒ | For null hypothesis testing, the test statistic (e.g. *F*, *t*, *r*) with confidence intervals, effect sizes, degrees of freedom and *P* value noted<br>*Give P values as exact values whenever suitable.* |
| ☒ | ☐ | For Bayesian analysis, information on the choice of priors and Markov chain Monte Carlo settings |
| ☒ | ☐ | For hierarchical and complex designs, identification of the appropriate level for tests and full reporting of outcomes |
| ☒ | ☐ | Estimates of effect sizes (e.g. Cohen's *d*, Pearson's *r*), indicating how they were calculated |

*Our web collection on statistics for biologists contains articles on many of the points above.*

## Software and code

Policy information about availability of computer code

| | |
|---|---|
| Data collection | LabScribe (iWorx), GROMACS 2021. |
| Data analysis | GraphPad Prism (version 8.0.0), OriginLab 2022, PyMol version 2.5.4 (Schodinger), UCSF ChimeraX version 1.5, MDAnalysis python package (version 2.0.0), matplotlib python package (version 3.8.2). |

For manuscripts utilizing custom algorithms or software that are central to the research but not yet described in published literature, software must be made available to editors and reviewers. We strongly encourage code deposition in a community repository (e.g. GitHub). See the Nature Portfolio guidelines for submitting code & software for further information.

## Data

Policy information about availability of data

All manuscripts must include a data availability statement. This statement should provide the following information, where applicable:

- Accession codes, unique identifiers, or web links for publicly available datasets
- A description of any restrictions on data availability
- For clinical datasets or third party data, please ensure that the statement adheres to our policy

All analysed TEVC data is presented in the main text or supplemental information. Representative whole cell traces are shown in Fig. S2. Raw data used to create box and whisker plots in figures 2a, 3d, and 5c is provided in a Source Data file. P values for statistical tests in each data table are also provided in the Source Data file. Primers used to create every mutant characterized in the manuscript are provided in a supplemental file. Initial and final frames from all simulations are

available (here). The following PDB entries were used in the manuscript: 2BG9 [https://doi.org/10.2210/pdb2BG9/pdb], 7QKO [https://doi.org/10.2210/pdb7QKO/pdb], 7QL5 [https://doi.org/10.2210/pdb7QL5/pdb], 6X3S [https://doi.org/10.2210/pdb6X3S/pdb], 6X3Z [https://doi.org/10.2210/pdb6X3Z/pdb], 7KOO [https://doi.org/10.2210/pdb7KOO/pdb], 7KOQ [https://doi.org/10.2210/pdb7KOQ/pdb], 6HIS [https://doi.org/10.2210/pdb6HIS/pdb], 6HIN [https://doi.org/10.2210/pdb6HIN/pdb], 6PXD [https://doi.org/10.2210/pdb6PXD/pdb], 6PLR [https://doi.org/10.2210/pdb6PLR/pdb], 3TLS [https://doi.org/10.2210/pdb3TLS/pdb], 5HEG [https://doi.org/10.2210/pdb5HEG/pdb], and 5HEW [https://doi.org/10.2210/pdb5HEW/pdb].

## Research involving human participants, their data, or biological material

Policy information about studies with human participants or human data. See also policy information about sex, gender (identity/presentation), and sexual orientation and race, ethnicity and racism.

| | |
|---|---|
| Reporting on sex and gender | N/A |
| Reporting on race, ethnicity, or other socially relevant groupings | N/A |
| Population characteristics | N/A |
| Recruitment | N/A |
| Ethics oversight | N/A |

Note that full information on the approval of the study protocol must also be provided in the manuscript.

# Field-specific reporting

Please select the one below that is the best fit for your research. If you are not sure, read the appropriate sections before making your selection.

☒ Life sciences ☐ Behavioural & social sciences ☐ Ecological, evolutionary & environmental sciences

For a reference copy of the document with all sections, see nature.com/documents/nr-reporting-summary-flat.pdf

# Life sciences study design

All studies must disclose on these points even when the disclosure is negative.

| | |
|---|---|
| Sample size | The sample size used throughout the manuscript refer to the number of individual experiments performed for each type of analysis. For TEVC experiments, a minimum of 8 oocytes were characterized for each mutant. The large sample size is used out of an abundance of caution to ensure that the reported mean is indicative of the most precise value. The relatively small error values associated with these means confirms the precision of the measurement. Mutants were characterized in oocytes from a minimum of two batches to ensure observed changes were not dependent on oocyte batch. Radiolabelled binding assays used to determine cell surface expression were performed on a minimum of three oocytes. The raw CPM values were normalized to WT and clear differences were observed for mutants that did not express vs those that did. The sample size was sufficient for a general assessment of "expressed" or "not expressed". Simulations were performed in triplicate, having broadly similar outcomes. Each converged and were replicable making the sample size sufficient. |
| Data exclusions | No data were excluded from the study. |
| Replication | All attempts are reproducibility were successful and can be gauged by the standard deviation within the data tables. See "Sample Size" for a measure of how often each experiment was repeated. n values for all experiments are reported in the data tables. |
| Randomization | Randomization is not applicable to this study as the study is not a clinical/drug trial. |
| Blinding | Blinding in not applicable to this study as the study is not a clinical/drug trial. |

# Reporting for specific materials, systems and methods

We require information from authors about some types of materials, experimental systems and methods used in many studies. Here, indicate whether each material, system or method listed is relevant to your study. If you are not sure if a list item applies to your research, read the appropriate section before selecting a response.

## Materials & experimental systems

| n/a | Involved in the study |
| --- | --- |
| ☒ | ☐ Antibodies |
| ☒ | ☐ Eukaryotic cell lines |
| ☒ | ☐ Palaeontology and archaeology |
| ☒ | ☐ Animals and other organisms |
| ☒ | ☐ Clinical data |
| ☒ | ☐ Dual use research of concern |
| ☒ | ☐ Plants |

## Methods

| n/a | Involved in the study |
| --- | --- |
| ☒ | ☐ ChIP-seq |
| ☒ | ☐ Flow cytometry |
| ☒ | ☐ MRI-based neuroimaging |
